# Cancer of the Liver, Pancreas and Peritoneum in Singapore

**DOI:** 10.1038/bjc.1961.4

**Published:** 1961-03

**Authors:** C. S. Muir


### CANCER OF THE LIVER, PANCREAS AND PERITONEUM IN SINGAPORE

C. S. MUIR

*From the Department of Pathology, University of Malaya in Singapore*

Received for publication November 4, 1960

THIS paper discusses, from two standpoints, malignant tumours of the liver, pancreas and peritoneum in Singapore. Firstly, the incidence and morbid anatomy of such tumours, seen in the post mortem room by the staffs of the University and Government Departments of Pathology, in the 22,997 autopsies carried out in the years 1948–58 inclusive, are described. Secondly, the morbidity and mortality from these cancers, derived from hospital and Registrar General's figures, for the years 1954–58 inclusive, are considered. This study is complementary to a previous review of gastro-intestinal tract tumours (Muir, 1959).

The age, sex, and racial structure of the Singapore population for this period have already been described (Muir, 1959) and some of the possible sources of error and bias in the post mortem material and in the records of the Registrar General (Singapore) have been indicated. Briefly, half the Singapore population, both living and post mortem, is under 21 years of age, and while the relative racial proportions for the population as a whole are : Chinese, 76·3 per cent ; Malay, 13·1 per cent ; Indians and Pakistanis, 8·1 per cent ; and Others 2·5 per cent, the corresponding post mortem figures are 87·0 per cent, 2·6 per cent, 8·4 per cent, and 2·0 per cent. Some one-third of deaths are registered by hospital assistants and police officers.

The records of nearly all post mortems performed on the Island in this period were available to the author. These were scrutinised individually and the relevant data entered on a proforma for subsequent analysis.

### LIVER

In all, there were 189 malignant tumours falling within the scope of International List No. 155, i.e., tumours of the ampulla of Vater, the biliary passages (both intra-hepatic and extra-hepatic), the gall bladder, and the liver itself.

Six of these were situated in the gall bladder. Five were in males. The mean age at death for the six was 58·2 $\pm$ 6·0 years. Spread to the liver and to the regional nodes was seen in 4 instances ; metastasis to the lungs, adrenals, peritoneum and stomach was noted in one case. Two of the livers showed cholangiectasis with abscess formation ; choleliathiasis was seen once.

A further five tumours, all in males, were found in the extra-hepatic bile ducts. The mean age at death was 46·8 $\pm$ 8·6 years. Two of these cancers arose in the common bile duct near the point of junction of the hepatic ducts, another in the left hepatic duct, and in the remaining two the exact point of origin was in doubt. Cholangiectasis was present in all five, calculi in four, biliary cirrhosis in two.

Secondary spread to the regional lymph nodes was seen in all five ; widespread metastasis to lung, liver, omentum, suprarenal, and heart, in two.

The remaining 178 were within the liver itself. One hundred and sixty-four of the tumours were found in males whose mean age at death was 48·6 ± 10·6 years, and 13 in females, the mean age at death being 44·5 ± 11·9 years. (A female aged 1 year is excluded from this figure.) The remaining cancer, a malignant haemangio-endothelioma, was discovered in a Chinese male aged 19 years.

Five of these primary liver tumours were seen in Indian males, whose mean age at death, 34·6 ± 8·1 years, was thus appreciably lower than that of the Chinese males.

The pattern of age distribution of the 178 cases is shown in Table I. It will

TABLE I.—*The Age Distribution, by Sex, of 178 Singapore Cases of Primary Liver Tumours*

| Age | Males | | Females | | Total | |
|---|---|---|---|---|---|---|
| | Number | Per cent | Number | Per cent | Number | Per cent |
| 0–9 | — | — | 1 | 8 | 1 | 1 |
| 10–19 | 3 | 2 | — | — | 3 | 2 |
| 20–29 | 5 | 3 | 1 | 8 | 6 | 3 |
| 30–39 | 18 | 11 | 3 | 23 | 21 | 12 |
| 40–49 | 64 | 39 | 4 | 31 | 68 | 38 |
| 50–59 | 47 | 28 | 2 | 15 | 49 | 28 |
| 60–69 | 25 | 15 | 1 | 8 | 26 | 15 |
| 70 + | 3 | 2 | 1 | 8 | 4 | 2 |
| Totals | 165 | 100 | 13 | 101 | 178 | 101 |

be noted that there were more deaths before the age of 50 than there were after. Liang and Tung (1959) tabulate the age distribution of primary liver cancers for three series ; 258 Chinese, 736 Europeans and Americans, and 569 Africans. In China 83 per cent of deaths occurred before the age of 50 ; in Europe and America, 32 per cent ; in Africa, 93 per cent. The peak incidence in China, as in Singapore, was in the decade 40–49.

Grossly, the vast majority of livers were enlarged. The mean weight for the male being 2800 g., for the female, 2300 g. The least weight recorded was 870 g., the greatest 7700 g. The tumours were found to be principally in the right lobe in 55 per cent, mainly in the left lobe in 21 per cent, being distributed more or less uniformly throughout in 21 per cent. The site was not stated in the remaining 3 per cent.

A solitary tumour was seen in 14 per cent. In 34 per cent satellite nodules of tumour were seen round the main mass, and in 20 per cent, as well as the principal tumour, nodules were found scattered throughout the remainder of the liver. In 29 per cent the nodules were multiple, no one being sufficiently larger than its fellows to warrant the title of principal tumour.

The solitary or principal tumours were usually about 12 cm. in diameter. They were generally soft and pulpy, green, reddish, and yellow-white in colour depending on the extent of necrosis, biliary impregnation and haemorrhage. The smaller nodules, 1 to 3 cm. in diameter, were usually white in colour, although a variegated appearance was seen from time to time. On occasion the nodules blended imperceptibly into the surrounding liver tissue. In some 5 per cent umbilication of small sub-capsular nodules was noted. If the main tumour mass reached the capsule, it tended to grow in an arboreal, or fern-like manner for some time beneath the capsule before penetration. Often the solitary or principal tumour protruded well beyond the liver, usually from the right lobe, down towards the pelvis. One such was 16 cm. × 12 cm. × 12 cm. in size.

Spread along the blood vessels of the liver was extremely common. In 24 per cent the portal vein was blocked with tumour as far as the junction of the major portal branches, and continued outside the liver to the lesser omentum in a further 22 per cent. The splenic vein was involved in 5 per cent, the mesenteric vein in 3 per cent and the gastric vein in 2 per cent. Doubtless due to the impeded venous return from the spleen, and in many, to a pre-existent cirrhosis, splenomegaly was common, being observed in 35 per cent. The largest spleen weighed 670 g., the mean weight being 340 g.

Spread along the hepatic veins was much less frequent, being found in 6 per cent. In a further 4 per cent the tumour extended into the inferior vena cava, and in another 2 per cent into the right atrium.

Metastasis within the abdominal cavity was infrequent. The diaphragm was involved, principally by direct extension, in 7 per cent ; the greater omentum in 6 per cent ; the lymph nodes of the porta hepatis, in 8 per cent : the para-aortic nodes in 5 per cent ; spread to the stomach was seen in 3 per cent ; to the kidney and adrenals in 2 per cent.

The lungs showed secondary tumour in 28 per cent. These were usually bilateral, very often sub-pleural, and of the cannon ball type. In some 4 per cent worm-like tumour casts could be expressed from the pulmonary arteries. Metastases were seen in bone and brain in 3 per cent of cases.

Oesophageal varices were noted in 15 per cent, and in 9 per cent one or more of these had ruptured with significant haemorrhage into the gut.

Ascites were present in 25 per cent, the majority showing at least a tinge of blood staining. Twenty-four per cent exhibited rupture of the tumour with consequent haemorrhage into the abdominal cavity. This varied considerably in amount, 1·5 litres being a not uncommon quantity. In the brief clinical histories accompanying the post mortem notes there was no mention of injury in any case, but in two persons the haemorrhage followed percutaneous aspiration biopsy.

Brown atrophy of the heart was noted in 15 per cent and seemed particularly common in those under 45 years of age.

The morbid anatomy corresponds, in the main, fairly closely to that of prior Singapore series of primary liver cancers (Tull, 1932 ; Shanmugaratnam, 1956), although Tull (1932) found portal vein invasion in but 20 per cent, and intra-abdominal haemorrhage in but one of 134 cases.

### THE PANCREAS AND PERITONEUM

There were 20 cases of pancreatic carcinoma. Fifteen were in Chinese males, whose mean age at death was 49·9 $\pm$ 7·5 years. Of the remainder, three were Chinese females, one an Indian male, one a Eurasian female. The mean age at death for the 20 was 52·5 $\pm$ 7·8 years.

The tumours were usually of the scirrhous type, rock-hard to the touch. Two were described as mucoid, one of which was a multiloculate cystadenocarcinoma. Thirty-five per cent were found in the head, 30 per cent encroached on both head and body, 20 per cent were in the body while 15 per cent extended over most of the organ.

Secondary spread to the lung was found in 30 per cent ; to the liver in 65 per cent ; to the brain, bones, and kidneys in 10 per cent. Peritoneal dissemination was seen in 20 per cent ; regional lymph node involvement in 70 per cent. The stomach and duodenum were directly invaded in 30 per cent while the extra-hepatic bile ducts were either narrowed or closed in only 30 per cent.

There were no primary tumours of the peritoneum at post mortem. Although 18 instances were registered these were probably secondary to malignancy elsewhere.

### MORTALITY AND MORBIDITY

The 178 primary liver neoplasms represent 0·77 per cent of all post mortems carried out in the 11 years under review, or, if the 11,525 children under the age of 11 years be omitted, 1·55 per cent, or, 16·2 per cent of the 1096 malignant tumours seen in the mortuary.

Liver cancer (List No. 155–56) accounted for 759 of the 5915 deaths from malignant disease recorded by the Registrar General (Singapore) in 1948–58, i.e. 12·8 per cent. In view of the post mortem prevalence of primary liver cancer the author had assumed that the bulk of these tumours would fall within List No. 155. By the kindness of the Singapore Government Department of Statistics, who provided, for 1957 and 1958, from their punched card records a more detailed breakdown of malignant tumours by site, sex, and five-year age group, this premise was shown to be false, only 13 per cent of all malignant liver tumours for the two years being entered under List No. 155. A closer scrutiny of this fuller analysis showed that, in fact, in 1957 and 1958 there were more primary carcinomata of the liver at autopsy (41) than there were registered (26). This would indicate a breakdown in the coding system.

With this in mind the grouping together of List No. 155 and 156, and the calculation of age specific death rates for cancer of the liver based on this aggregate, was considered justified. These are given in Table II. Those for 1957 are related to the 1957 census population, and those for 1957 and 1958 combined are again related to the same population, the larger numbers so obtained tending to smooth out any irregularity of age distribution from year to year. For comparison similar rates are given for malignant neoplasms as a whole (List No. 140–205).

If it be assumed that there were, in fact, few secondary liver cancers, then in 1954–58 primary liver neoplasms were nearly as common as bronchial carcinomata, and about half as frequent as gastric cancers. In the 11-year post mortem review the relative proportions of primary cancers of the liver, lung and stomach were 1·00 : 0·92 : 0·97 ; in 1954–58 the proportions were 1·00 : 1·2 : 1·07.

In the eleven years there were 839 admissions to hospital with a liver cancer (List No. 155–156) of whom 354, or 42 per cent, died there. With 189 post mortems the autopsy rate was thus 53 per cent.

For the years 1948–53 inclusive the hospital returns included carcinoma of the pancreas under " other digestive organs ". From 1954 onwards it has been entered as a disease in its own right under International List No. 157. In the quinquennium 1954–58 there were 64 admissions to hospital with 28 deaths.

In the same period of time the Registrar General (Singapore) recorded 37 deaths ; 13 (35 per cent) of the cases registered in the five years came to post mortem. The crude incidence rate for both sexes is 0·5 per 100,000 per annum. In view of the small numbers, age specific death rates are not given ; however, an age adjusted figure is to be found in Table III.

The crude death rate from cancer (List No. 140–205) in the quinquennium 1954–58 was 51·6 per 100,000 living per annum, from liver cancer (List No. 155–156) 6·3 per 100,000, and from malignancies of the digestive organs and peritoneum (List No. 150–159) 25·0 per 100,000. Thus these latter cancers comprised 48·5 per cent of all malignant tumours registered. Virtually comparable figures, for England and Wales, Japan and Australia, are given by the World Health Organization (1956). In the triennium 1951–53, for males these were 46·0, 85·1, and 49·5 per cent ; for females, 44·9, 60·4 and 43·3 per cent. In Singapore, in 1957–58, 55·8 per cent of male malignancies registered, and 38·5 per cent of the female, arose in the digestive system and peritoneum.

It is held that the comparison of tumour incidence between countries is facilitated by the use of age adjusted death rates. Such adjustments are usually made to the population of the writer's own country. Segi (1960) has derived a standard population based on the age constitution, in 1950, of 46 widely scattered countries, and it is to this hypothetical standard population that adjustment has been made in Table III. It will be noted that as in Table II, a figure has been given for Singapore, for 1957, and for the mean incidence in 1957–58, both based on the 1957 census population. The values for England and Wales, Japan, and Australia, based on the same standard population, are taken from Segi (1960). The figures speak for themselves.

This type of comparison may be criticised if the figures for a relatively small population are adjusted to those of a much larger. To circumvent this in some measure, the mean number of cases observed in the Singapore populace in 1957–58 is compared with the number expected for the populations of England and Wales, Japan, and Australia (Table IV). In the calculation of these expected numbers,

TABLE II.—*Age Specific Death Rates, per* 100,000 *Living per annum, for Liver Cancer* (*List No.* 155–156) *and for All Cancers* (*List No.* 140–205)

| | Liver cancer | | | | | | All cancer | | | | | |
|---|---|---|---|---|---|---|---|---|---|---|---|---|
| | 1957 | | | 1957–58 | | | 1957 | | | 1957–58 | | |
| Age | Male | Female | Total | Male | Female | Total | Male | Female | Total | Male | Female | Total |
| 0–4 | — | — | — | — | — | — | 7·3 | 5·5 | 6·4 | 5·9 | 5·1 | 5·5 |
| 5–9 | — | — | — | — | — | — | 2·7 | 3·8 | 3·2 | 3·1 | 2·8 | 3·0 |
| 10–14 | — | — | — | 0·7 | — | 0·4 | 4·2 | — | 2·2 | 7·7 | 3·1 | 5·5 |
| 15–19 | 1·4 | — | 0·7 | 0·7 | — | 0·4 | 11·4 | 4·6 | 8·1 | 9·3 | 3·8 | 6·6 |
| 20–24 | — | — | — | — | — | — | 3·3 | 3·4 | 3·3 | 5·7 | 4·3 | 5·0 |
| 25–29 | 3·3 | 3·8 | 3·6 | 2·5 | 1·9 | 2·2 | 10·0 | 9·6 | 9·8 | 16·7 | 14·4 | 15·7 |
| 30–34 | — | — | — | 7·0 | — | 3·9 | 21·9 | 30·2 | 25·6 | 25·9 | 22·6 | 24·5 |
| 35–39 | 12·9 | — | 7·3 | 8·6 | 2·7 | 6·0 | 48·3 | 55·2 | 50·8 | 50·5 | 60·8 | 55·0 |
| 40–44 | 11·6 | 3·0 | 7·9 | 17·4 | 1·5 | 10·5 | 83·6 | 93·4 | 87·9 | 94·1 | 85·9 | 90·5 |
| 45–49 | 45·0 | 3·4 | 27·0 | 45·0 | 6·9 | 28·4 | 185·3 | 120·9 | 157·4 | 202·5 | 127·8 | 170·1 |
| 50–54 | 51·2 | 17·7 | 36·7 | 49·6 | 8·9 | 31·9 | 263·2 | 199·6 | 235·5 | 261·5 | 179·7 | 225·9 |
| 55–59 | 48·7 | 11·8 | 32·0 | 48·6 | 23·7 | 37·4 | 384·4 | 266·2 | 331·1 | 403·9 | 248·4 | 333·7 |
| 60–64 | 83·2 | 48·9 | 65·9 | 74·9 | 40·8 | 57·7 | 532·7 | 269·1 | 399·6 | 566·0 | 289·5 | 426·4 |
| 65–69 | 147·9 | 46·3 | 90·9 | 110·9 | 23·1 | 61·7 | 739·4 | 301·0 | 493·5 | 613·7 | 330·0 | 454·6 |
| 70–74 | 29·1 | 37·3 | 34·2 | 43·8 | 46·7 | 45·6 | 554·7 | 355·2 | 433·1 | 729·9 | 345·9 | 495·8 |
| 75 + | 46·3 | — | 14·5 | 46·3 | — | 14·5 | 463·2 | 253·8 | 319·4 | 625·3 | 285·5 | 392·0 |

TABLE III.—*Comparison of Age Adjusted Death Rates for Cancers of the Liver (List No.* 155–156) *and Pancreas (List No.* 157) *and for All Cancers (List No.* 140–205

| Country | Liver | | | All cancer | | | Pancreas | | |
|---|---|---|---|---|---|---|---|---|---|
| | Male | Female | Total | Male | Female | Total | Male | Female | Total |
| Singapore, 1957 | 17·62 | 5·78 | 11·77 | 112·8 | 79·1 | 90·8 | 1·29 | 0·46 | 0·69 |
| Singapore, 1957–58 | 16·98 | 5·22 | 11·21 | 121·2 | 73·5 | 94·9 | 1·41 | 0·71 | 1·00 |
| England and Wales, 1956–57 | 3·38 | 3·09 | — | 170·9 | 113·4 | — | 6·36 | 4·01 | — |
| Japan, 1956–57 | 15·20 | 9·36 | — | 127·6 | 94·0 | — | 2·18 | 1·49 | — |
| Australia, 1956–57 | 3·57 | 3·63 | — | 130·9 | 100·4 | — | 6·14 | 3·51 | — |

TABLE IV.—*Comparison of Number of Cases Observed in the Singapore Population with the Number Expected for the Populations of England and Wales, Japan and Australia. (For details see Text)*

| | Liver | | All cancer | | Pancreas | |
|---|---|---|---|---|---|---|
| | Male | Female | Male | Female | Male | Female |
| England and Wales | 10·4* | 10·2 | 574·0* | 420·4* | 19·5* | 12·6* |
| Japan | 52·5* | 32·4 | 452·3 | 361·8* | 8·8 | 5·7 |
| Australia | 11·8* | 11·9 | 415·7* | 365·5* | 18·9* | 11·2* |
| Singapore | 78·0 | 20·0 | 498·0 | 299·0 | 5·0 | 2·5 |

the age specific death rates by 5-year group, for these three countries for 1956–57, as given by Segi (1960) were linked with the Singapore 1957 census population. On the assumption that cancer is a rare event occurring in a large population, and that the numbers observed will follow a Poisson distribution, significant differences, at a probability level of 0·005 (single tail) are indicated by an asterisk (*). The limits of the expectation are taken from Fisher and Yates (1957).

Bearing in mind the limitations of the basic data, cancer of the liver (List No. 155–156) is significantly commoner in Singapore males than in the male populace of England and Wales, Japan, and Australia, whereas in the female there is no such difference. Cancer (List No. 140–205) is more frequent in the male of England and Wales. Pancreatic tumours are more prevalent in both sexes in England and Wales, and Australia, than in Singapore.

Comparison with earlier years is always of interest, despite differences in the presentation and grouping of data.

In 1907–12 a series of 121 post mortems on cancer patients was carried out at Tan Tock Seng's Hospital, Singapore. This institution was at that time a free hospital for male adult paupers, principally of Chinese race. 25·6 per cent of these tumours were in the liver (Hoffman, 1915).

Hoffman (1935) analysed mortality from 1028 various cancers in Singapore for the years 1926–31. This study is particularly valuable, despite the broad groupings of the various tumours, as a racial breakdown was given. Using these figures, and the population data of the April 1931 census, when in a population of 555,745 individuals 75 per cent were Chinese, 12 per cent were Malaysians, 9 per cent were Indians or Pakistanis, Europeans and Eurasians each 1 per cent and Others 2 per cent, the mean annual rates for cancers of the stomach and liver per 100,000 persons per annum were respectively 23·1, 8·9, 13·0, 12·4, 29·0, and 24·1, i.e., a mean incidence of 20·4. In 1948–58 the incidence of these malignancies was 15·5 per 100,000 population, reflecting, not a decrease in incidence, but a vast increase in the number of children on the Island.

At this time the male/female ratio for these tumours was 4·4 to 1 and carcinomata of the liver and stomach accounted for 55·4 per cent of all malignant tumours, whereas in 1948–58 they comprised 34·4 per cent.

Tull (1932) found 134 cases of primary liver cancer in a total of 17,664 autopsies carried out at Tan Tock Seng Hospital, Singapore, an incidence of 0·76 per cent. This figure is rather lower than might be expected in a free hospital for male adult paupers. The 90 per cent autopsy rate and the higher death rate at this time from tropical disease possibly accounts for this. Using data supplied by Tull, Bonne (1937) noted that in Singapore, the liver was the primary site in 36 per cent of 128 post mortems on cancer cases.

In Malaya (1925–31) Hoffman (1935) found that 46·8 per cent of the 1493 deaths ascribed to malignancy were caused either by gastric or by hepatic neoplasms. In the same septennium these tumours accounted for 24·8 per cent of 1780 cancer cases treated in hospital.

In view of the paucity of post mortems on Malays, the biopsy data collated by Marsden (1958) from 4650 malignant tumours submitted for opinion to the Institute for Medical Research, Kuala Lumpur, are of significance. 7·5 per cent of all malignant neoplasms in Chinese, 3·0 per cent in Indians, and 2·7 per cent in Malays, arose in the liver (List No. 155), this despite the reluctance of Malays to seek hospital treatment, let alone submit to biopsy. Marsden (1958) calculates the apparent incidence rates for Chinese and Indians to be 6·2 and 2·2 per 100,000 living per annum respectively (Singapore mean, for all races, for List No. 155–156, 6·3 per 100,000) and remarks that the incidence in Malays is probably as high as in Chinese.

In 1948–57 there were 2032 admissions to Malayan hospitals with liver cancers (List No. 155–156), 46 per cent dying from the disease, a morbidity of 2·5 per 100,000 per annum (Singapore : 6·4 per 100,000 per annum).

The Registrar General (Malaya) does not give a detailed breakdown of deaths in his reports, rightly considering that to do so would be misleading as only 20 per cent of deaths are certified by medical practitioners.

There seems little doubt that liver cancer is very frequent in South-East Asia.

Vedder (1927) in a post mortem series on 157 Filipino cancer cases found that 39 per cent of tumours arose in the liver or gall bladder. Kouwenaar (1951) found in Java, on analysis of the post mortem records of a group of estate hospitals, that of 120 Chinese males who died from some form of malignant disease, in 31 per cent the primary tumour was hepatic. In 59 Javanese males the proportion was much higher, 78 per cent. These figures are astonishingly high, and are even more remarkable when one realises that these figures represent respectively 2·8 and 3·9 per cent of all the necropsies performed. While it is likely that this population was a selected one, Kouwenaar (1951) does not state its composition.

Nguyen-Van-Ai (1958) has described the malignant tumours in South Vietnamese based on biopsy material. Of 1303 malignant neoplasms only 6 per cent were from the digestive tract, and but 0·8 per cent arose in the liver. In Thailand, again in biopsies, 5 per cent of 350 malignant tumours were considered hepatic in origin (Vellios, Goonchorn, and Suvanatemiya, 1953).

Cooray (1954) found 4 liver cell tumours, 2 tumours of the gall bladder and bile ducts, and 2 tumours of the pancreas in 57 post mortems on cases of malignant disease at Colombo, Ceylon. In 1815 malignant biopsies corresponding figures were 9, 1 and 2. Cooray (1951) points out that too much reliance cannot be placed on these figures as post mortems are few in number.

Mitra and Gupta (1960) have attempted to estimate the overall incidence of cancer in India by applying death registration figures for the City of Calcutta to the sub-continent. In the Calcutta corporation area no corpse can be disposed of without a death certificate from a competent medical officer, although this often means a certificate based merely on the reported history of a case after death has taken place. In a total of 1645 deaths from malignancy, 276; or 16·7 per cent, were due to liver tumours. The male/female ratio was 1·4 : 1. The peak incidence, 36 per cent of cases, was in the age group 60 and over. If these figures hold good for the whole of India, as the authors suggest they may, then each year there must be, in round figures, 30,000 new cases of liver cancer. Based on an estimated population of 380 million, of an age structure not unlike that in Singapore, the crude mortality rate for these tumours must be in the order of 7 to 8 per 100,000, a value not far removed from the Singapore estimate of 6·3 per 100,000.

Despite these vast numbers, in 1957 Thompson, Hackley, and McGinnis, in a review of the world literature were able to collect only 2205 cases, a reflection of the vast pool of material still to be tapped in the Orient.

A perusal of the above data forces one to the conclusion that in South-East Asia there is but one territory where figures of morbidity and mortality from malignant disease can be usefully compared with those from elsewhere, i.e., Singapore.

In the West, hepatic carcinomata comprise about 0·12 per cent of all post mortems and about 1·2 per cent of all carcinomata (Büngeler and Eder, 1960). Extremely high figures have been reported from isolated centres such as Tobolsk in Soviet Siberia where cholangiocarcinomata, associated with opistorchiasis, comprised 3 per cent of all autopsies (Kraft, 1958).

As the majority of tumours in this paper were in Chinese, data from China are of great interest. From January 1950 to June 1957, 21,706 post mortems were carried out in 38 medical colleges. One thousand nine hundred and seventy-nine malignancies were encountered (9·1 per cent), and of these 262 (13·2 per cent) were primary liver tumours, i.e., 1·2 per cent of all necropsies. The incidence in hospital admissions varied from 0·07 to 0·3 per cent. Most of these liver tumours were seen in persons between the ages of 31 to 50, the mean age being 40 (Yeh, 1959).

Yeh and Cowdry (1954) report that in 1025 autopsies carried out in Formosa (Taiwan) there were 140 instances of malignant disease (13·7 per cent) of which 28 (20 per cent) arose in the liver.

The biological behaviour of liver tumours has attracted some attention. Shanmugaratnam (1956) states that cholangiocarcinoma seems to be equally prevalent in both East and West, and it is only the hepatocellular tumours which are the more numerous in the Orient. Reviewing the literature he finds that bile duct tumours are more frequent in females, unlike the liver cell variety, and that extra-hepatic metastases are frequent and widespread.

In addition to the differentiation between hepatocellular and cholangiocellular tumours, various attempts have been made to further classify the tumours arising in the parenchymal liver cells. Liang and Tung (1959) divided their 38 cases into two major types, the massive and the nodular, these occurring in their material with roughly equal frequency. Almost all of the massive tumours were situated in the right lobe, with or without smaller satellite nodules. Tumour thrombi in the portal vein were rarely seen, whereas lung metastases were common. This type of tumour was usually found with a Laennec type of cirrhosis. Death was due either to cachexia or to intra-abdominal rupture of the main tumour mass.

The nodular tumours they further separated into mononodular and multinodular. Four of five of their cases of mononodular carcinomata were situated along the Sereje-Cantlie line, i.e. from gall bladder to inferior vena cava. Generally livers with nodular carcinoma were lighter in weight than the massive type. The portal veins were frequently involved whereas pulmonary secondaries were relatively rare. Mononodular tumours were associated with post-necrotic scarring, the multinodular with Laennec's cirrhosis. Death usually followed haemorrhage from either ruptured oesophageal varices or from intra-abdominal rupture of the tumour.

Histological differences were tentatively suggested : mitotic figures and tumour giant cells were more frequent in massive tumours, stromal fibrosis being commoner in the nodular type.

The authors do not give figures for some of their observations, using terms like " frequent, rare, relatively rare, etc." in comparison. Analysis of the given figures reveals one statistically significant difference ($P < 0{\cdot}01$) : an increased prevalence of lung metastases in the massive tumours.

Using these criteria, an attempt was made to classify the present material. This was not easy and more reliance was placed on the photographs accompanying Liang and Tung's (1959) communication, than on the textual description. Division into mononodular and multinodular types was not possible. With these reservations 60 per cent of tumours fell into the massive category, 33 per cent into the nodular, and 7 per cent could not be classified.

Ruptured oesophageal varices were found in 5 per cent of massive tumours, and in 17 per cent of the nodular. Corresponding figures for intra-abdominal haemorrhage following rupture of the tumour were 20 per cent and 35 per cent. The differences are significant ($P < 0{\cdot}05$). There were fewer lung metastases, and more varices, with more frequent invasion of the portal vein, in the nodular than in the massive, but the differences are not significant. On average, the nodular livers were 170 g. lighter.

The figures for this series are thus in broad consonance with those of Liang and Tung (1959) from Canton, although the division is by no means clear-cut.

Shanmugaratnam (1956) found a disparity between the immigrant Chinese from South China and the indigenous Chinese of Singapore, who are of the same ethnic origin, in their susceptibility to develop liver cell carcinoma. Now that new immigration from China has ceased, and that fewer Singapore Chinese return to China for extended holidays, if his hypothesis that there is a liver cancer inducing environmental factor present in China, and absent in Singapore, is correct, then his analyses over the next thirty years, as the China-born Chinese die off, will be of great interest.

There is some evidence that the incidence of liver cell cancer is higher in South China than in the North (Liu, 1953), a difference which has been ascribed to Clonorchiasis. Chlonorchiasis and schistosomiasis were rare in the present series : neither parasite is found in Malaya or Singapore. Yeh (1959) quoting Lin (1958)

states that of 537 cases of primary liver cancer reported in Chinese literature in the past 10 years only 10 showed a concomitant schistosomal infection, and that in these there appeared to be no relationship between the ova and the growth. The significance of *Clonorchis sinesis* infection has been extensively investigated by Hou (1956).

### SUMMARY

The morbid anatomy of the 178 primary malignant neoplasms of the liver seen at autopsy in Singapore in the period 1948–58 inclusive is presented.

The age and sex incidence are tabulated. The peak incidence is in the fifth decade, more than half the deaths taking place before the age of 50.

In the post mortem room cancers of the liver, stomach, and lung are seen in virtually equal numbers, but in the returns of the Registrar General (Singapore) the respective ratios of incidence are 1 : 2 : 1. The crude mortality rate, based on registered deaths, from liver cancer (List No. 155–156) is 6·3 per 100,000 living per annum.

Age-specific death rates for cancer (all sites) and for cancer of the liver are given for 1957 and 1957–58.

The incidence of cancer (all sites), and of cancer of the liver and pancreas is compared with that of England and Wales, Japan, and Australia, by derivation of age-adjusted death rates, and by calculation of expected numbers.

Cancer of the liver is seen to be significantly more prevalent in the Singapore male, while cancer (all sites) is significantly commoner in the male of England and Wales.

Tumours of the pancreas and peritoneum are briefly mentioned. In both sexes pancreatic tumours are shown to be significantly less common than in England and Wales, and Australia.

A division of the liver tumours into massive and nodular types, as proposed by Liang and Tung (1959), is attempted, and some of the differences in biological behaviour noted by these writers are tentatively confirmed.

A portion of the relevant literature from South-East Asia, India, and China is discussed, and the importance of Singapore as a centre for epidemiological research in this part of the Orient is emphasized.

I wish to thank Professor R. Kirk, Dr. K. Shanmugaratnam, Senior Pathologist, Singapore, and Dr. J. Higginson for much helpful advice and criticism, Mr. S. C. Chua, Chief Statistician, Singapore, and his staff for their help, and Mr. P. A. Samuel who typed the script. This paper forms part of a thesis for the degree of Ph.D. (Malaya).

### REFERENCES

Bonne, C.—(1937) *Amer. J. Cancer*, **30**, 435.

Büngeler, W. and Eder, M.—(1960) *Dtsch. med. Wschr.*, **85**, 959.

Cooray, G. H.—(1951) *J. Ceylon Br. Brit. med. Ass.*, **46**, 7.—(1954) *Acta Un. int. Cancr.*, **10**, 31.

Fisher, R. A. and Yates, F.—(1957) ‘ Statistical Tables for Biological, Agricultural and Medical Research ’. Edinburgh (Oliver and Boyd), p. 61.

Hoffman, F. L.—(1915) ‘ The Mortality from Cancer throughout the World ’. Newark, New Jersey (The Prudential Press), p. 712.—(1935) *Amer. J. Cancer*, **24**, 661.

HOU, PAO-CHANG.—(1956) *J. Path. Bact.*, **72**, 239.
KOUWENAAR, W.—(1951) *Docum. neerl. indones. Morb. trop.*, **3**, 357.
KRAFT, I. A.—(1958) *Vop. Onkol.*, **4**, 321.
LIANG, PO-CH'IANG AND TUNG, CHÜN.—(1959) *Chin. med. J.*, **79**, 336.
LIN, T.—(1958) *Chin. J. Path.*, **4**, 52.
LIU, Y.—(1953) *Chin. med. J.*, **71**, 183.
MARSDEN, A. T. H.—(1958) *Brit. J. Cancer*, **12**, 161.
MITRA, S. AND GUPTA, A. D.—(1960) *Bull. World Hlth Org.*, **22**, 485.
MUIR, C. S.—(1959) *Brit. J. Cancer*, **13**, 595.
NGUYEN-VAN-AI.—(1958) *Bull. Soc. Pat. exot.*, **51**, 421.
SEGI, M.—(1960) 'Cancer Mortality for Selected Sites in 24 Countries (1950–1957)'. Sendai (Tohoku University).
SHANMUGARATNAM, K.—(1956) *Brit. J. Cancer*, **10**, 232.
THOMPSON, D. J., HACKLEY, D. J. AND MCGINNIS, C. M.—(1957) *J. chron. Dis.*, **5**, 205.
TULL, J. C.—(1932) *J. Path. Bact.*, **35**, 557.
VEDDER, E. B.—(1927) *J. Amer. med. Ass.*, **88**, 1627.
VELLIOS, F., GOONCHORN, S. G. AND SUVANATEMIYA, P.—(1953) *Cancer*, **6**, 188.
WORLD HEALTH ORGANIZATION.—(1956) *Epidem. vit. Stat. Rep.*, **9**, 316.
YEH, SHU AND COWDRY, E. V.—(1954) *Cancer*, **7**, 425.
YEH, TUAN-FU.—(1959) *Chin. med. J.*, **79**, 538.


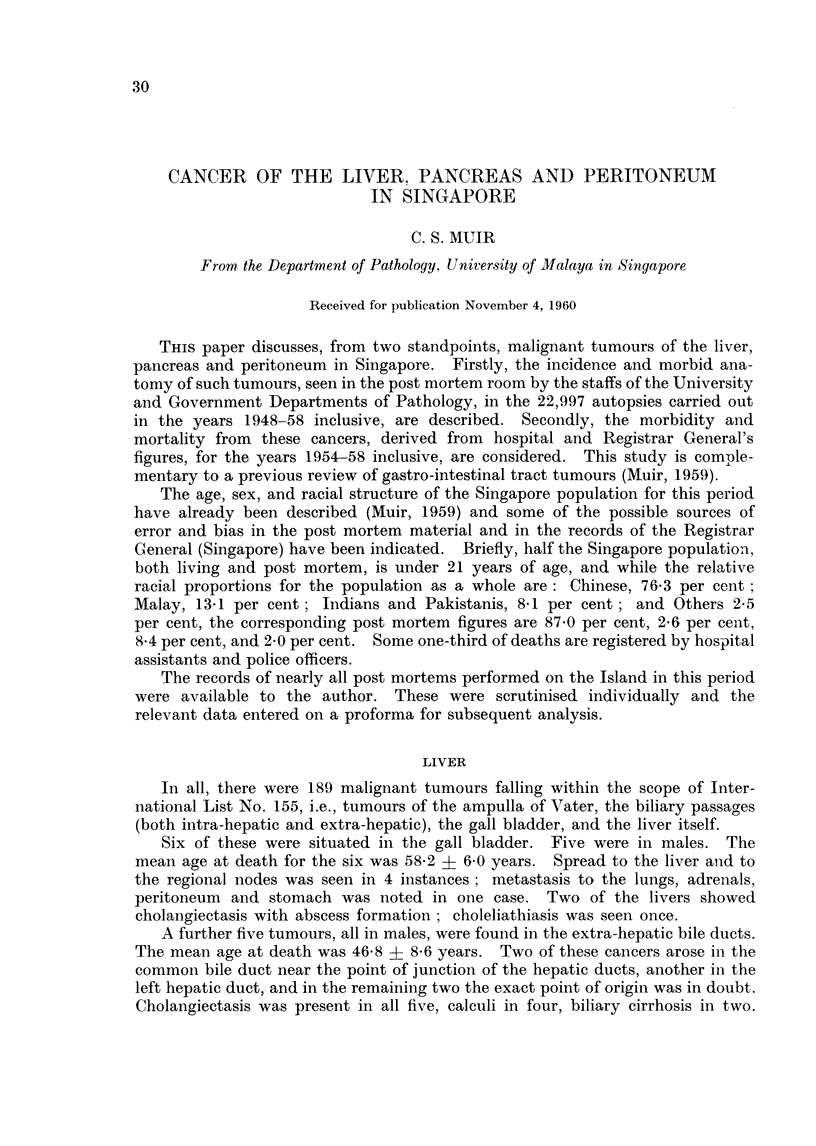

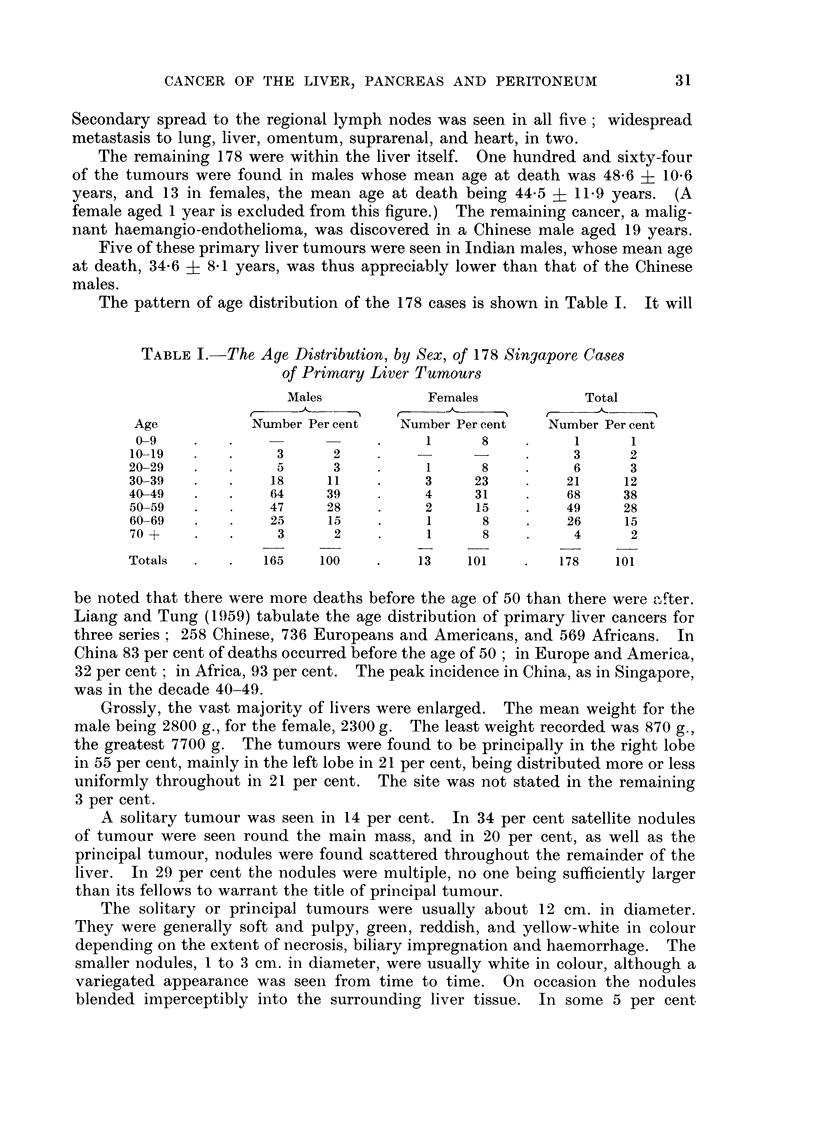

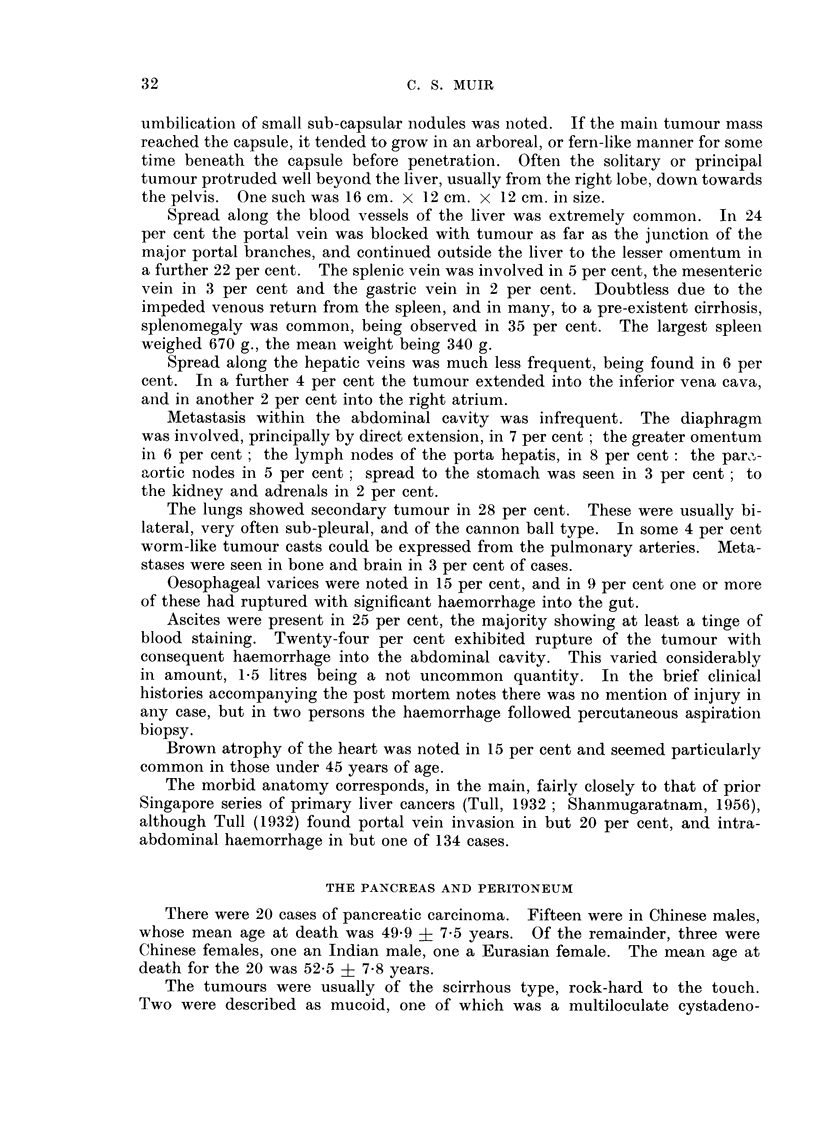

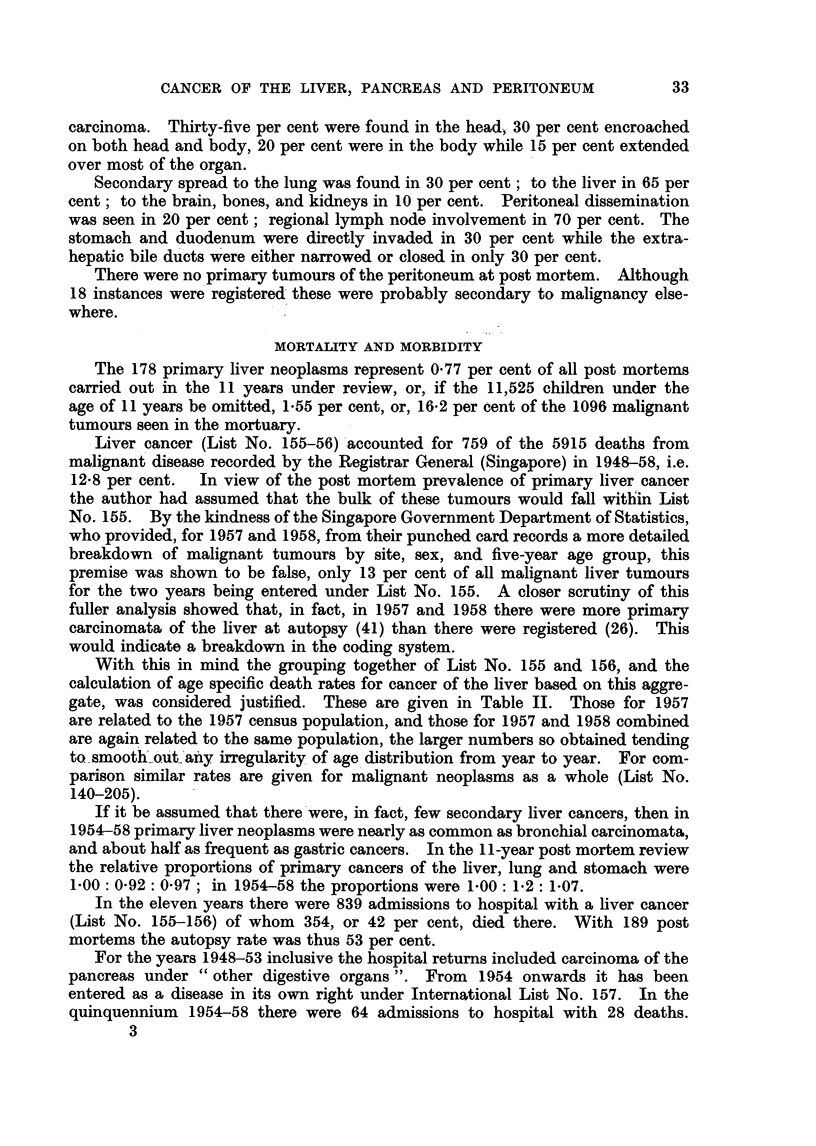

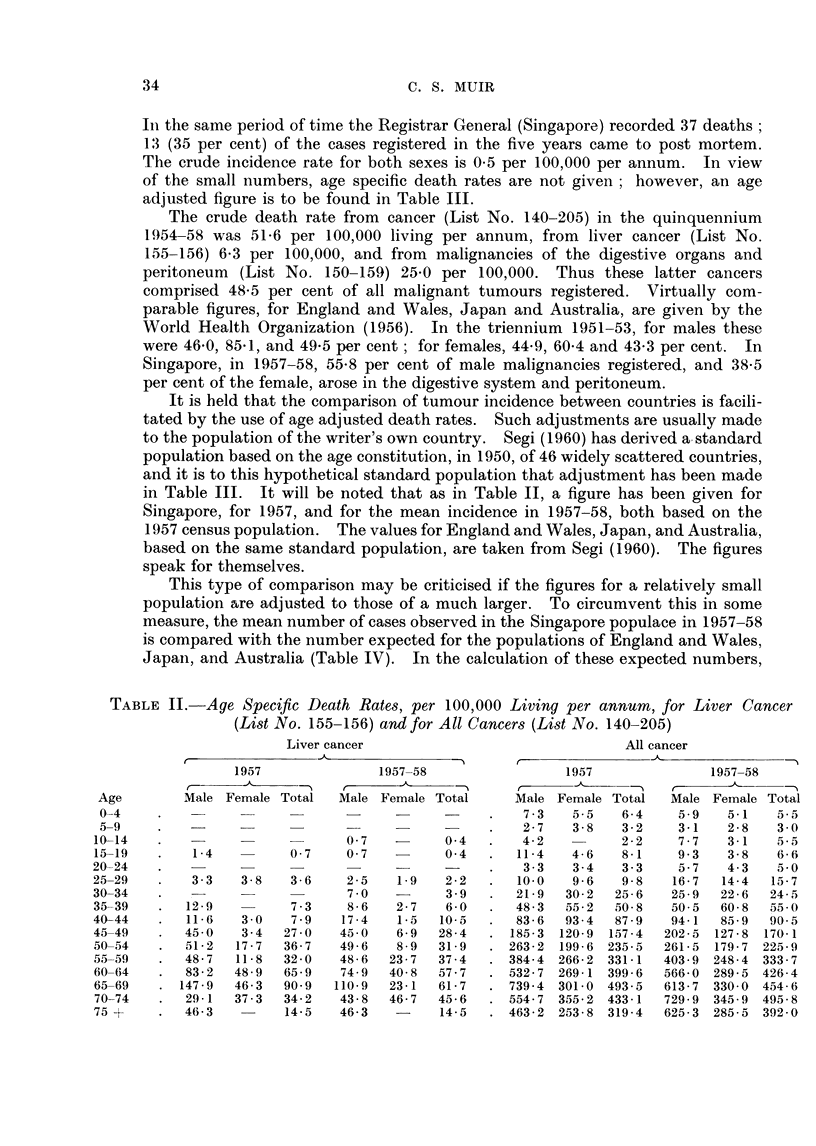

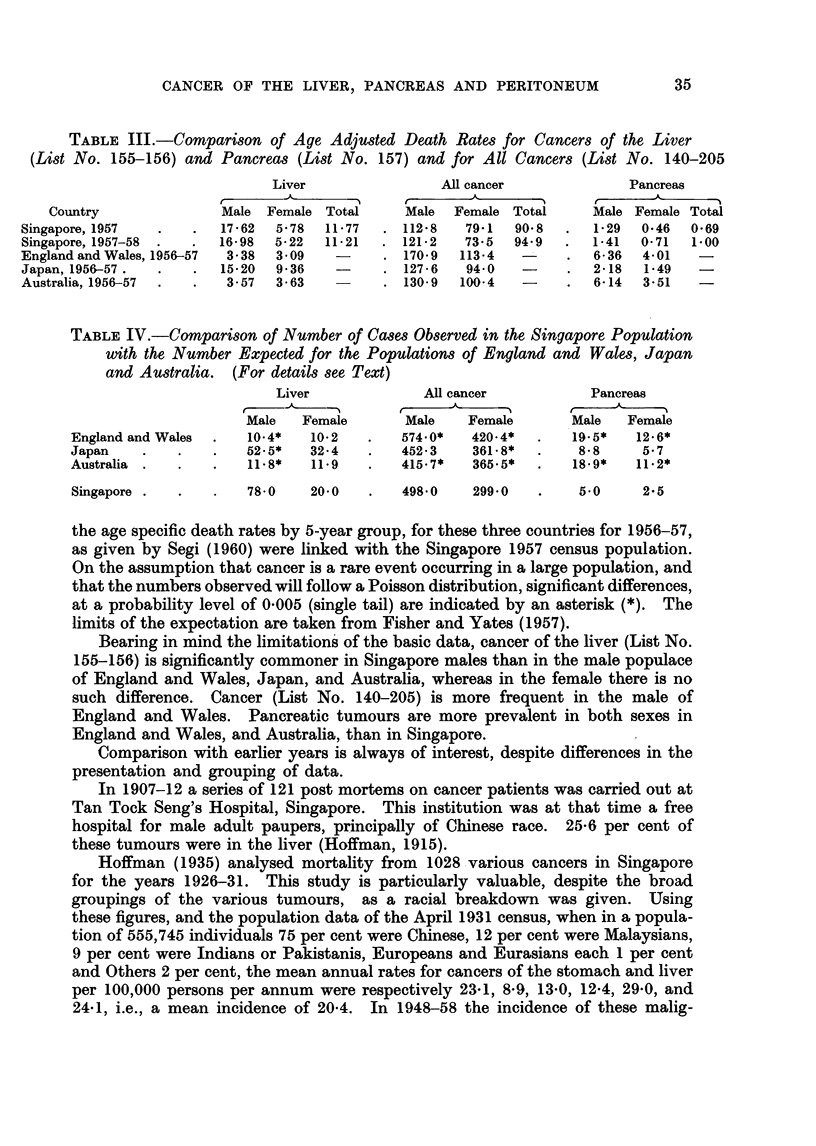

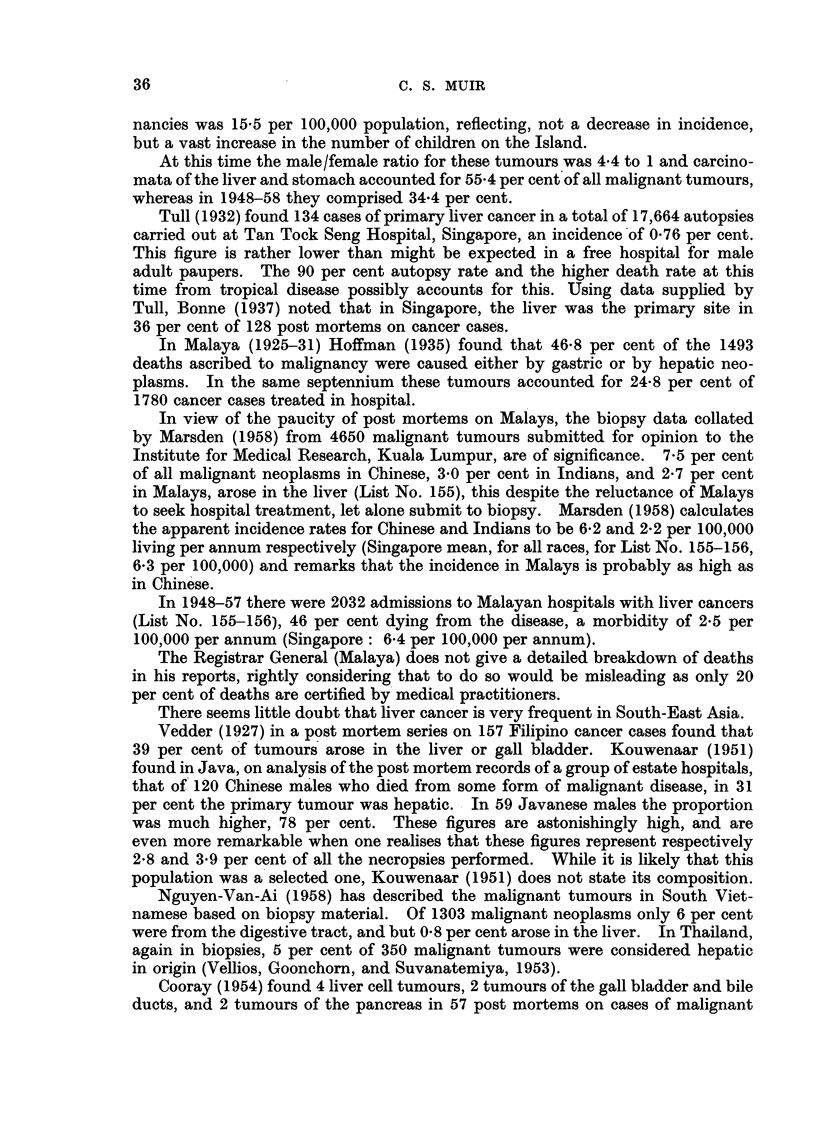

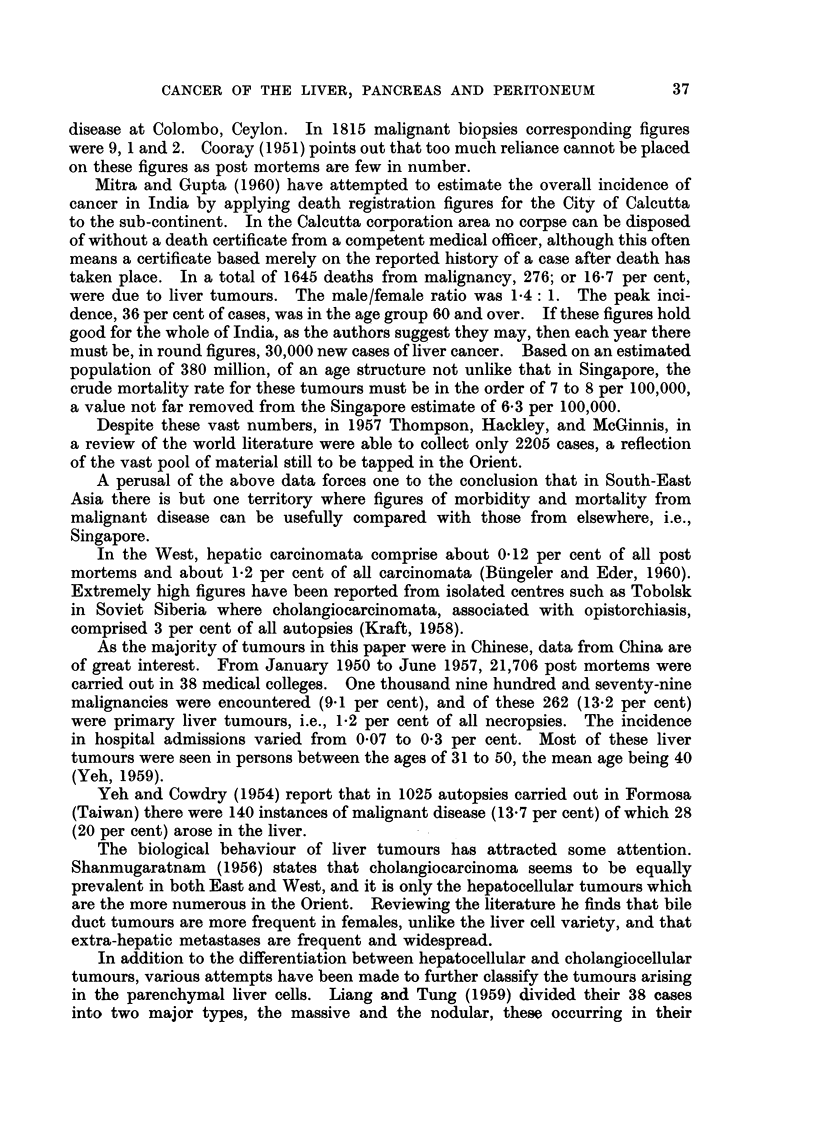

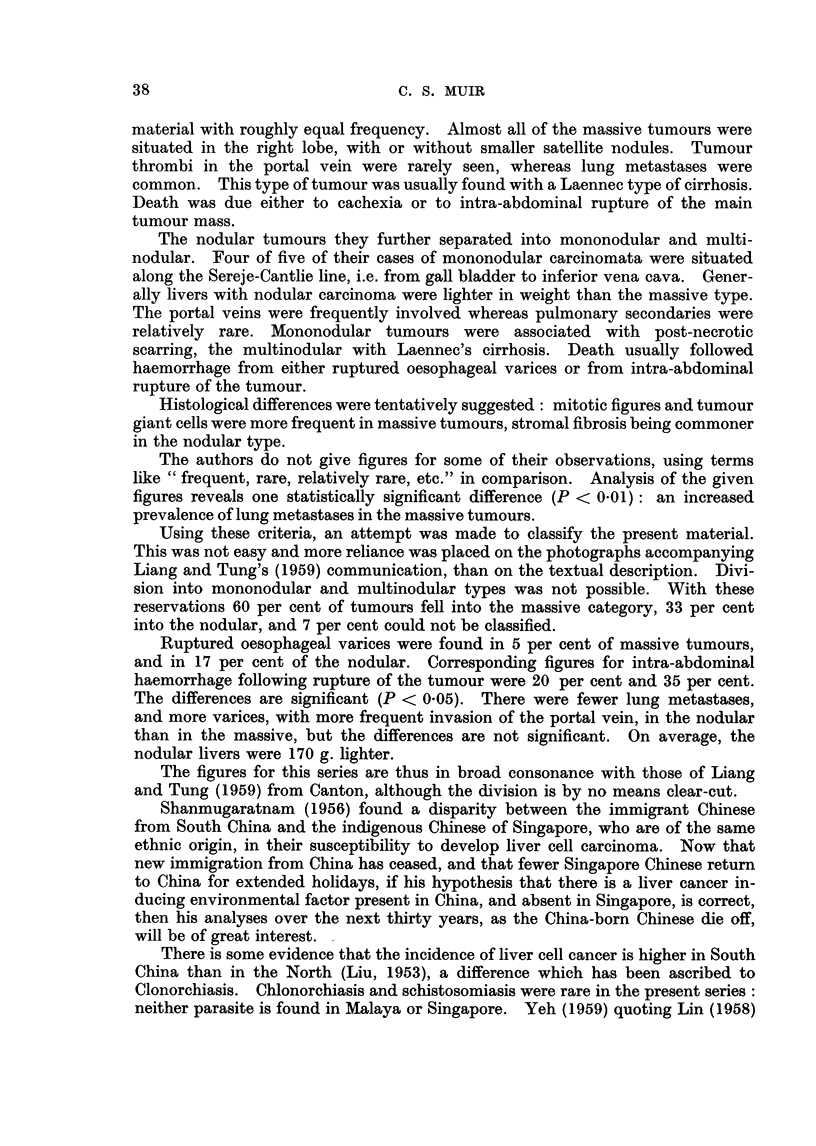

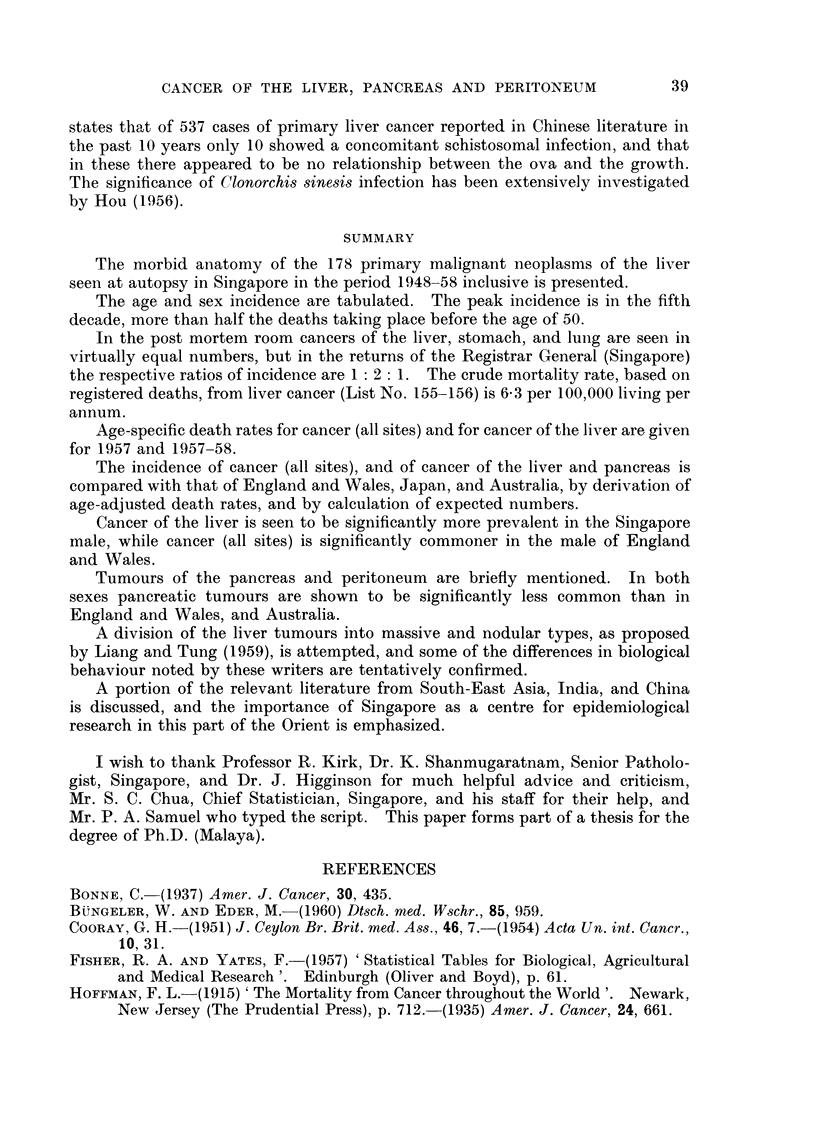

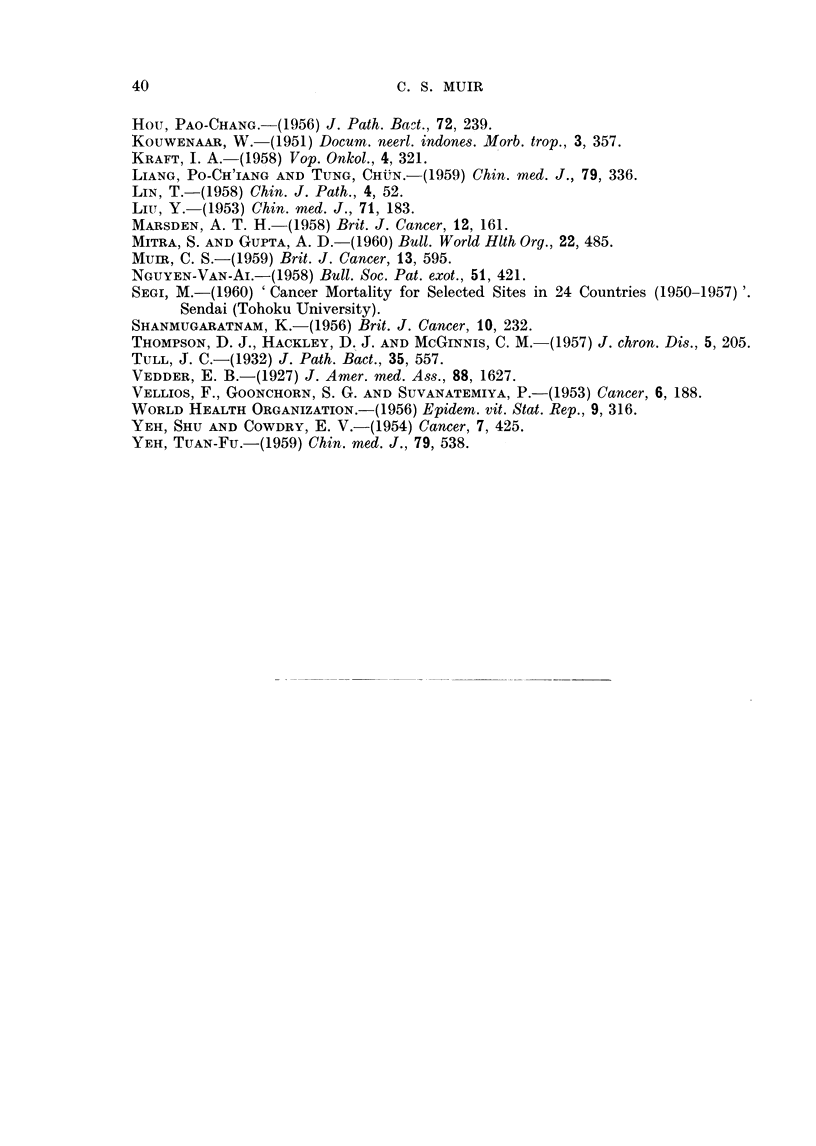


## References

[OCR_00641] BUENGELER W., EDER M. (1960). [Primary liver carcinoma].. Dtsch Med Wochenschr.

[OCR_00660] KRAFT Ia (1958). Osobennosti i chastota pervichnogo raka pecheni v Tobol'ske.. Vopr Onkol.

[OCR_00662] LIANG P. C., TUNG C. (1959). Morphologic study and etiology of primary liver carcinoma and its incidence in China.. Chin Med J.

[OCR_00669] MITRA S., DAS GUPTA A. (1960). An estimate of the prevalence of cancer in India.. Bull World Health Organ.

[OCR_00674] NGUYEN-VAN-AI (1958). Les cancers au sud-Viet-nam en 1957; fréquences absolue et relative; répartition selon le type histologique, le siège, l'age, le sexe, la race; comparaison avec les années précédentes.. Bull Soc Pathol Exot Filiales.

[OCR_00678] SHANMUGARATNAM K. (1956). Primary carcinomas of the liver and biliary tract.. Br J Cancer.

[OCR_00680] THOMPSON D. J., HACKLEY D. J., MCGINNIS C. M. (1957). Primary carcinoma of the liver; review of the literature and an analysis of twelve additional cases.. J Chronic Dis.

[OCR_00687] YEH S., COWDRY E. V. (1954). Incidence of malignant tumors in Chinese, especially in Formosa.. Cancer.

[OCR_00688] YEH T. F. (1959). Primary carcinoma of the liver.. Chin Med J.

